# Supplementary material for: Higher-order correlations reveal complex memory in temporal hypergraphs
Source: Nat Commun. 2024 Jun 4;15:4754. doi: 10.1038/s41467-024-48578-6 (PMC11150504; doi:10.1038/s41467-024-48578-6)
Supplement: Supplementary file 1 — Supplementary Information [file 41467_2024_48578_MOESM1_ESM.pdf]

Supplementary Information of the manuscript
Higher-order correlations reveal complex memory in temporal hypergraphs

Luca Gallo,[1] Lucas Lacasa,[2] Vito Latora,[3,4,5,6] and Federico Battiston[1]

[1] *Department of Network and Data Science, Central European University, Vienna, Austria*
[2] *Institute for Cross-Disciplinary Physics and Complex Systems (IFISC), CSIC-UIB, Palma de Mallorca, Spain*
[3] *School of Mathematical Sciences, Queen Mary University of London, London E1 4NS, UK*
[4] *Department of Physics and Astronomy, University of Catania, 95125 Catania, Italy*
[5] *INFN Sezione di Catania, Via S. Sofia, 64, 95125 Catania, Italy*
[6] *Complexity Science Hub Vienna, A-1080 Vienna, Austria*

INTRA-ORDER CORRELATIONS IN SOCIAL SYSTEMS WITH HIGHER-ORDER INTERACTIONS

In the main text, we focused on a dataset describing face-to-face interactions in a scientific conference. In this section, we present the analysis of the intra-order temporal correlations for three other social systems. Two of the datasets come from the Sociopatterns project, and they describe face-to-face interactions *(i)* in an office, among $N = 92$ workers, over 11 days [1]; and *(ii)* in a hospital ward, among $N = 75$ patients, doctors, nurses, and administrative staff, over 72h [2]. The third dataset contains instead proximity data of $N = 692$ students at the campus of the Technical University of Denmark (DTU) in Copenhagen, for a period of one month [3].

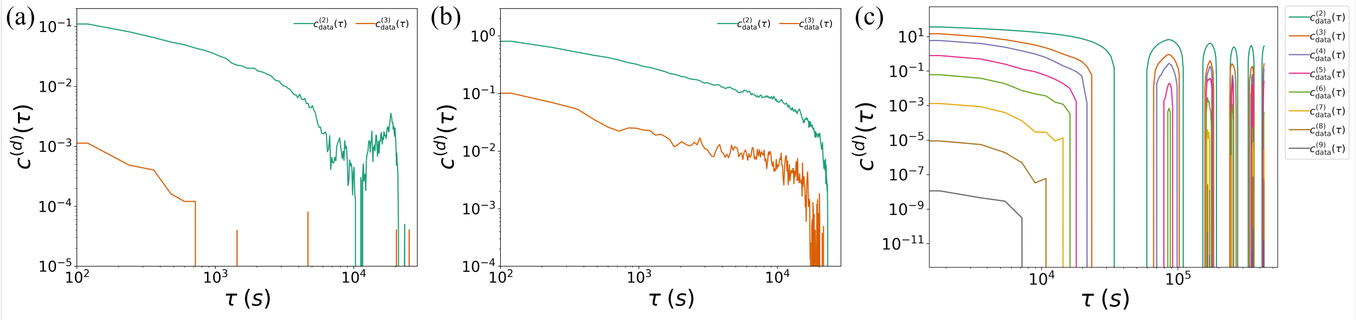

FIG. S1. Intra-order correlation functions in human face-to-face interactions in the office (a), in the hospital ward (b), and in the university campus (c), for different group sizes.

In this section, we analyze temporal correlations within groups of the same size. Fig. S1 depicts the values of the intra-order correlation functions for several group sizes d, for the three empirical systems examined, namely face-to-face interactions in the workplace (a), the hospital ward (b), and in the university campus (c). For each system, we consider only those group sizes for which we have a sufficient statistics. In general, for each social system, a hierarchy of long-range, slowly-decaying temporal autocorrelations emerges across different group sizes. Also, we note that intra-order correlations are lost abruptly after a typical time threshold that decreases with the group size d, i.e., larger groups remain temporally correlated for shorter time.

We observe periodicity patterns in the interactions occurring in the workplace and in the university campus. For this latter in particular, we note a series of peaks at large timescales that show the same hierarchical structure observed at small timescales. See Fig. S13 for a minimal model of a temporal hypergraph with periodicity.

CROSS-ORDER CORRELATIONS SHOW NONTRIVIAL TEMPORAL ORGANIZATION OF HIGHER-ORDER INTERACTIONS

In this section, we deepen our analysis on temporal correlations between groups of different sizes. In the main text, we showed that statistically significant cross-order correlations emerge. In particular, we focused on the cross-order correlations between groups of sizes four and five in the face-to-face interactions in the scientific conference. We here show that other pairs of group sizes in the same system are temporally correlated.

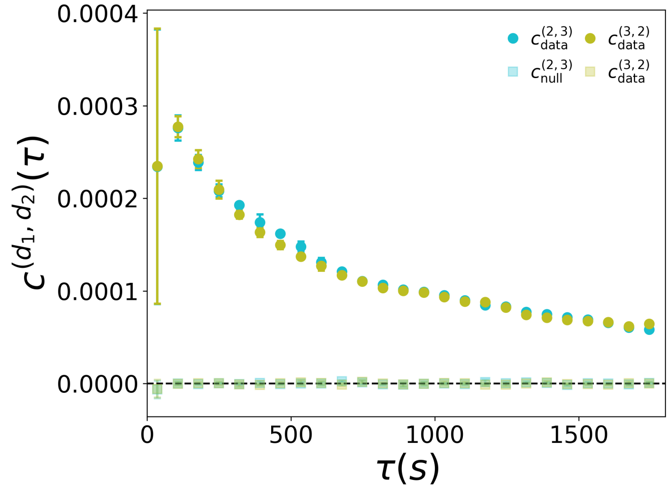

FIG. S2. Cross-order correlation functions for groups of sizes two and three, i.e., $c^{(2,3)}(\tau)$ (cyan) and $c^{(3,2)}(\tau)$ (olive), in the face-to-face interactions in the scientific conference. The empirical system (dark circles) is compared with a randomized null model with reshuffled time-steps (light squares). The values of the cross-order correlation functions are binned averaged, with the error bars representing the standard deviation.

Fig. S2 displays the cross-order correlation functions $c^{(2,3)}(\tau)$ (cyan) and $c^{(3,2)}(\tau)$ (olive), describing cross-order correlations between groups of sizes two and three. We also report the cross-order correlation functions for a randomized null model where correlations have been removed by time-reshuffling (lighter squares) Both functions exhibit an exponential decay that does not match the trends of the cross-order correlation functions $c^{(4,5)}(\tau)$ and $c^{(5,4)}(\tau)$ shown in Fig. 2(a) of the main text, suggesting that different pairs of group sizes can show different kinds of interdependencies.

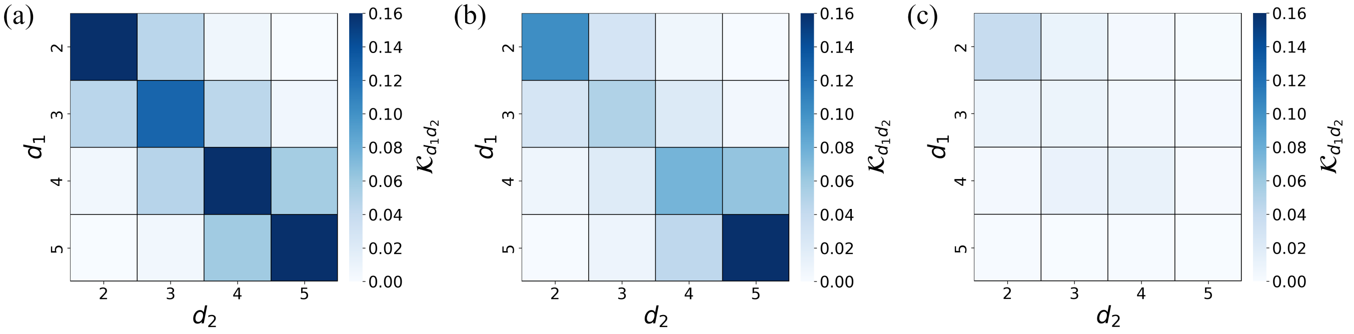

FIG. S3. Normalized cross-order interaction matrix $\mathcal{K}(\tau)$ at time lags $\tau = 60s$ (a), $\tau = 300s$ (b), and $\tau = 1500s$ (c), in the face-to-face interactions in the scientific conference.

Fig. S3 displays the normalized interaction matrix $\mathcal{K}(\tau)$ at different time lags, namely $\tau = 60s$ (a), $\tau = 300s$ (b), and $\tau = 1500s$ (c). We observe that cross-order temporal correlations can emerge between different group sizes. In particular, as discussed in the main text, the matrix $\mathcal{K}(\tau)$ shows a banded structure around the main diagonal, meaning that correlations between groups of similar sizes is higher. The banded structure is lost at large time lags, as correlations vanish at larger timescales.

The banded structure of the matrix $\mathcal{K}(\tau)$ suggests that, in the scientific conference dataset, groups tend to change

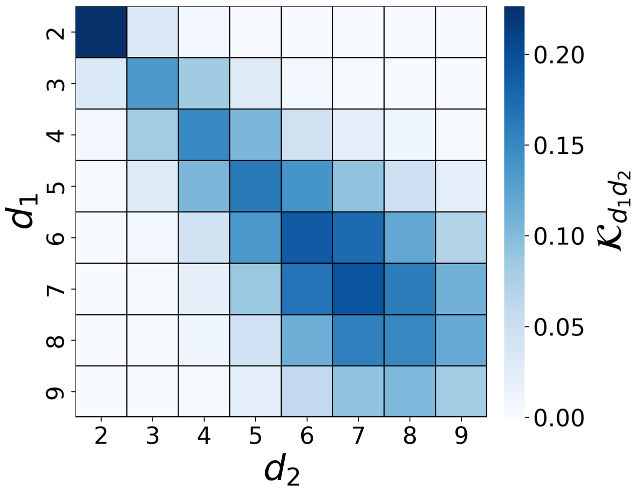

FIG. S4. Normalized interaction matrix $\mathcal{K}(\tau)$ at time lag $\tau = 300s$, in the DTU campus in Copenhagen.

gradually, with the loss or the addition of one member. We now want to investigate whether groups of larger size display the same property. To answer this question, we focus on the interactions among students occurring in the DTU campus in Copenhagen, in which larger groups have a sufficient statistics. Fig. S4 displays the normalized interaction matrix $\mathcal{K}(\tau)$ at time lag $\tau = 300s$. Once again, the matrix $\mathcal{K}(\tau)$ reveals a characteristic banded structure. However, compared to the scientific conference case, we note that groups of larger size show a different pattern. Indeed, we can observe significant cross-order correlations between groups of dissimilar sizes, e.g., groups of size nine are temporally correlated with groups of size five. This result suggests that larger groups evolve differently compared to smaller ones. In particular, while small-size groups change gradually, losing or gaining one member at a time, large-size groups show more complex dynamics, as they can also split in smaller groups or emerge from them.

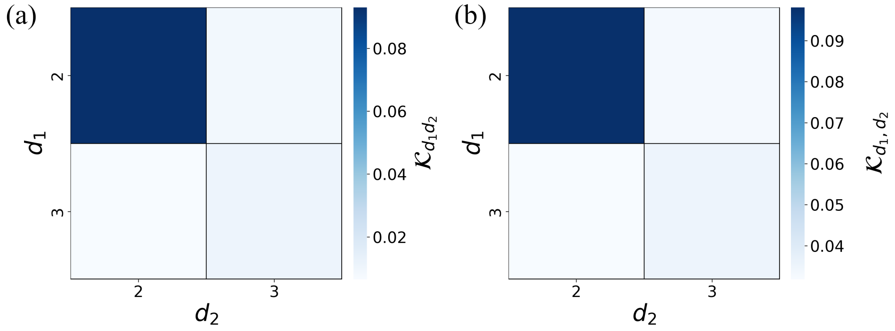

FIG. S5. Normalized interaction matrix $\mathcal{K}(\tau)$ at time lag $\tau = 300s$ for the face-to-face interactions occurring in the office (a) and in the hospital ward (b).

For completeness, in Fig. S5 we report the normalized interaction matrices evaluated at time lag $\tau = 300s$ relative to the interactions in groups of size two and three occurring in the office (a) and in the hospital ward (b).

In the main text, we showed that the cross-order correlation functions relative to groups of sizes d_1 and d_2 can differ significantly, as groups of a given size can anticipate those of another one, while the vice versa might not be true, or the magnitude of the two effects might be distinct. This discrepancy between the two functions is quantified in terms of the cross-order gap function $\delta^{(d_1,d_2)}(\tau)$. To complement the analysis provided in the main text, here we consider the interactions in the scientific conference, studying the possible gaps in the cross-order correlation functions for other pairs of group sizes.

Fig. S6 shows the cross-order gap function $\delta^{(d_1,d_2)}(\tau)$ calculated for each couples of orders of interaction (d_1, d_2). Note that the figure axis scales are the same as those of Fig. 2(c) of the main text (reported as panel (f) in the Figure), for a proper comparison (note that panel(d) has the same scale but focuses on the negative values of $\delta^{(d_1,d_2)}$). The results obtained for the empirical system (purple circles) are compared with those of a null model where the time-steps

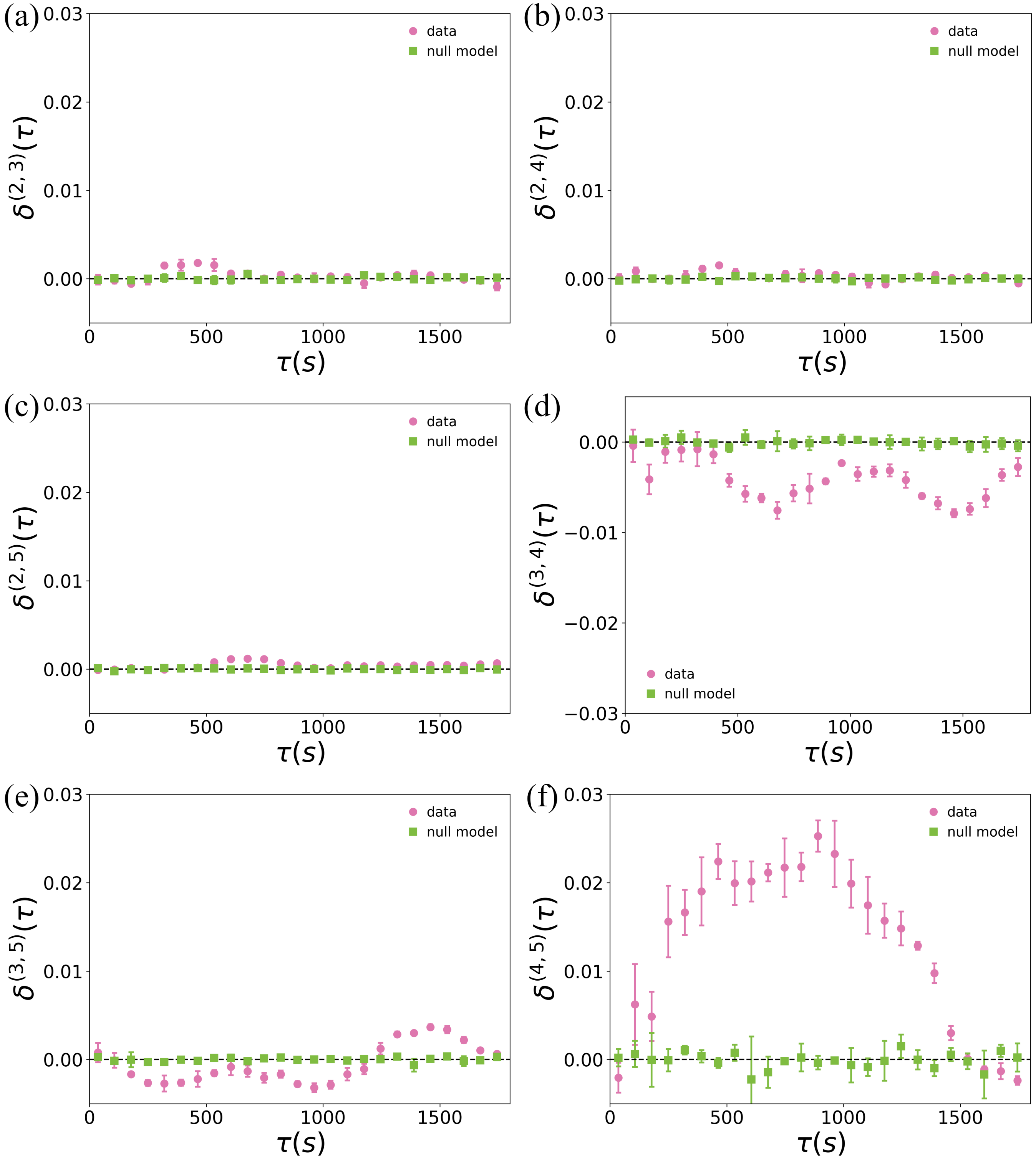

FIG. S6. Cross-order gap functions $\delta^{(d_1,d_2)}$ for the scientific conference data set (purple circles) and a null model (green squares). Panels (a) to (f) show the cross-order gaps for different pairs of orders up to $d = 5$. The values of the cross-order gap functions are binned averaged, with the error bars representing the standard deviation.

are reshuffled (green squares). We observe a variety of patterns in the cross-gap functions across different couples of orders. For instance, $\delta^{(2,3)}$, $\delta^{(2,4)}$ and $\delta^{(2,5)}$ do not significantly differ from the null model where temporal snapshots

are reshuffled. This suggests that the groups of size neither anticipate groups of sizes three, four and five, nor come after them, indicating a symmetry between the aggregation and the disaggregation processes. Instead, we observe significant differences in the cross-order correlations between groups of sizes three and four, i.e., $\delta^{(3,4)}$, and between groups of sizes three and five, i.e., $\delta^{(3,5)}$. Regarding, the first, we observe that $\delta^{(3,4)}$ is significantly negative in almost the entire range of the time lag τ, meaning that the formation of a group of four individuals from a group of three is less probable than the loss of one member in groups of four individuals. In other words, we observe a preferred temporal direction in the dynamics of group formation/fragmentation. However, compared to the case discussed in the main text, i.e., the cross-order gap between groups of sizes four and five, in which aggregation was more probable than the disaggregation, in this case we observe the opposite behavior. A mixed scenario, where group formation is more probable for certain values of τ, while group fragmentation is more probable for other lags, is shown in panel (e), representing the cross-order gap function $\delta^{(3,5)}$. In particular, disaggregation from a groups of size five to groups of size three seems to be more probable for smaller values of τ, i.e., after short time, while aggregation becomes more probable for larger values of τ, namely after long time.

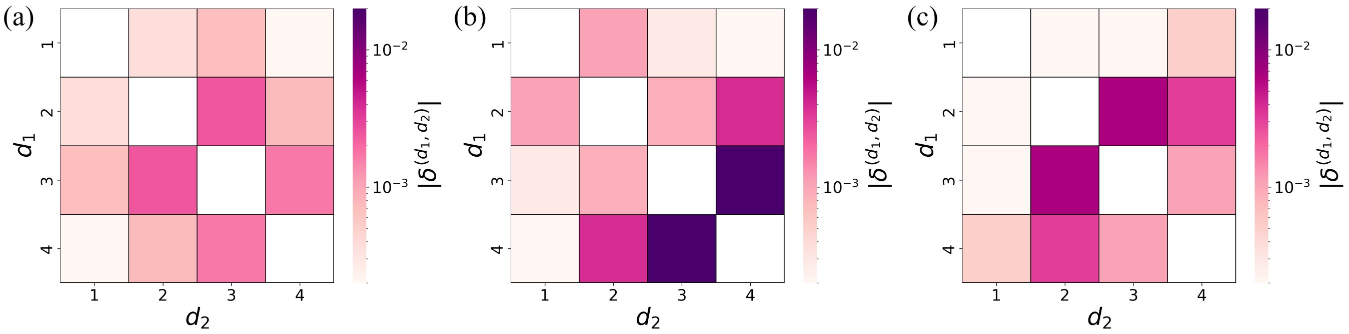

FIG. S7. Value of $|\delta^{(d_1,d_2)}|$ for different group sizes d_1 and d_2, at different times lags, namely $\tau = 60s$ (a), $\tau = 600s$ (b), and $\tau = 1500s$ (c), in the face-to-face interactions in the scientific conference.

Fig. S7 shows the value of $|\delta^{(d_1,d_2)}|$ for different values of d_1 and d_2, at different times lags, namely $\tau = 60s$ (a), $\tau = 600s$ (b), and $\tau = 1500s$ (c). We observe that different other pairs d_1, d_2 display a non-zero value of $|\delta^{(d_1,d_2)}|$, again suggesting a non-trivial temporal organization between different orders of interactions.

ANALYSIS OF THE DARH MODEL

In this section we analyze the Discrete Auto-Regressive Hypergraph (DARH) model, namely a minimal model of temporal higher-order network with memory. As described in the main text, in the DARH model, each hyperedge h^α evolves independently and at each time step t it updates its state either by drawing from its memory or randomly. The draw from the past occurs with probability $q^{(d)}$, with $d \in \{2, \dots, D\}$ for hyperedges of sizes two up to D, while with probability $1 - q^{(d)}$, the next state is chosen at random, according to a Bernoulli process with probability $y^{(d)}$. When the state of the hyperedge is drawn from the past, we assume hyperedges of size d to copy one of the previous $m_s^{(d)}$ states. Formally, the dynamics of a hyperedge of order d, h^α, is given by

$$h^\alpha_{t+1} = Q_t h^\alpha_{t-\mu} + (1 - Q_t) Y_t, \tag{S1}$$

where $Q_t \sim \text{Bernoulli}(q^{(d)})$, $Y_t \sim \text{Bernoulli}(y^{(d)})$, and $\mu \sim \text{Uniform}(1, m_s^{(d)})$.

We generate a temporal hypergraph using the DARH model. We build a higher-order network of $N = 5$ nodes, composed by $T = 10^5$ snapshots. We consider hyperedges up to order four. The memory lengths for the interactions of orders two, three and four are set to $m_s^{(2)} = 5$, $m_s^{(3)} = 4$, and $m_s^{(4)} = 3$, respectively. The probabilities of drawing from the past for the different orders of interaction are set to $q^{(2)} = 0.60$, $q^{(3)} = 0.55$, and $q^{(4)} = 0.50$. Finally, the probabilities to activate each hyperedge in the memory-less process are set to $y^{(2)} = 0.4$, $y^{(3)} = 0.05$, and $y^{(4)} = 0.02$. Fig. S8 shows the intra-order correlation functions for each order of interaction (a) and the cross-order correlation functions $c^{(2,3)}(\tau)$ and $c^{(3,2)}(\tau)$ (b) for the temporal hypergraph generated with the DARH model. For simplicity, for the cross-order correlation functions we limited the analysis to hyperedges of orders two and three.

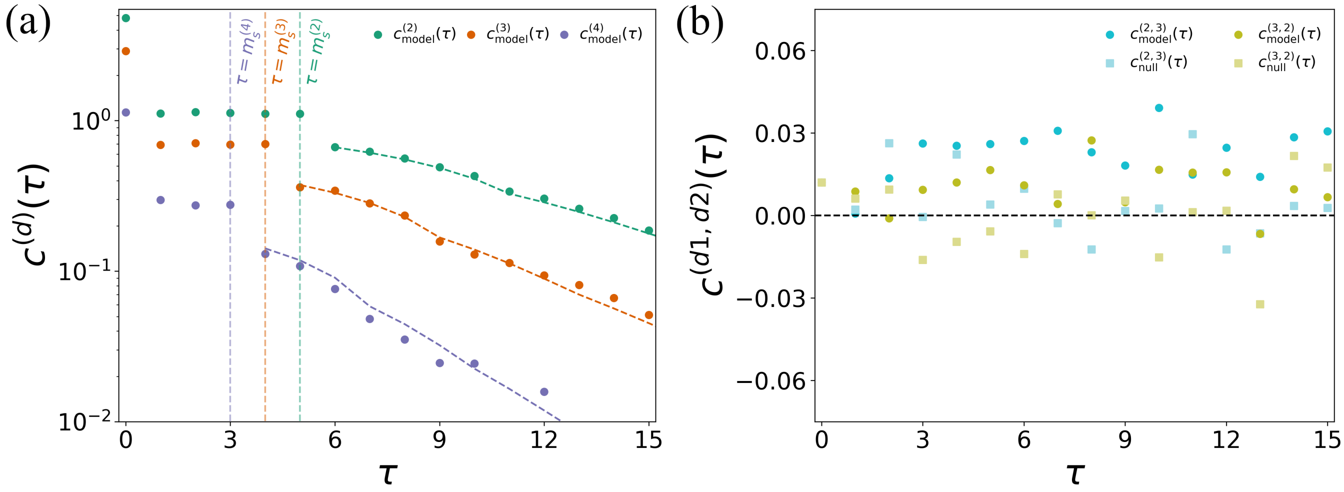

FIG. S8. Intra-order correlation functions and cross-order gap functions in the DARH model. (a) Intra-order correlations. Each color represents a different order of interaction. Vertical dashed lines represent the values $\tau = m_s^{(d)}$, with $d \in 1, 2, 3$. Other colored dashed lines display the theoretical prediction of the Yule-Walker equations. (b) Cross-order correlation functions between hyperedges of orders two and three, namely $c^{(2,3)}(\tau)$ (cyan) and $c^{(3,2)}(\tau)$ (olive). Dark dots represent the values obtained for the DARH model, while light squares show the correlation functions for a time-reshuffled hypergraph.

In Fig. S8(a), we observe that for all hyperedge orders the intra-order correlation remain constant for $1 \leq \tau \leq m_s^{(d)}$ (vertical dashed lines), while it decays exponentially after that value. At $\tau = 0$ the value of the autocorrelation functions is trivially higher, and depends on the average number of hyperedges of a given order over the whole temporal range.

For each order d, the intra-order correlation function can be reproduced by the Yule-Walker equations (dashed lines):

$$c^{(d)}(\tau) = \frac{q^{(d)}}{m_s^{(d)}} \sum_{k=1}^{m_s^{(d)}} c^{(d)}(\tau - k), \tag{S2}$$

which determine the autocorrelation function of a DAR($m_s^{(d)}$) process. In particular, one can prove [4] that $c^{(d)}(\tau)$ is constant for $\tau \le m_s^{(d)}$, with its value given by

$$c^{(d)}(\tau \le m_s^{(d)}) = c^{(d)}(0) \left[m_s^{(d)} \left(\frac{1}{q^{(d)}} - 1 \right) + 1 \right]^{-1}, \tag{S3}$$

which allows us to solve Eqs. (S2) up to any finite lag τ. The DARH model highlights that memory can be a driving factor for the emergence of intra-order temporal correlations. The model reveals that the hierarchical structure of the correlation ranges observed in the data (see both main text and previous sections of the Supplementary information) is due to the different degrees of memory possessed by the hyperedges.

Fig. S8(b) shows instead that hyperedges of sizes two and three do not display cross-order correlation. Indeed, both $c^{(2,3)}(\tau)$ (cyan) and $c^{(3,2)}(\tau)$ (olive) are close to zero (dark dots), and do not differ significantly from the values obtained for a null model where the time-steps of the temporal hypergraph have been reshuffled (light squares). Indeed, in the DARH model each hyperedge evolves independently from other hyperedges, either those having the same size and those of different sizes. As a consquence, we observe no cross-order correlations among hyperedges of different sizes.

So far we have assumed that a hyperedge of order d can update its state by copying one of its previous $m_s^{(d)}$ states. Therefore, the value of $m_s^{(d)}$ only depends on the order d of the interaction, meaning that each hyperedge of the same order has the same memory length. Such an assumption can be too restrictive, as each hyperedge can have in principle a different degree of memory. We relax this assumption by sampling the intra-order memory length of a hyperedge h_α of order d from a uniform distribution, i.e., $m_s^{(d)}(h_\alpha) \sim \text{Uniform}(0, m_{s,\max}^{(d)})$. Compared to the simplest version of the model, where we can set the value of the intra-order memory length $m_s^{(d)}$ for each order of interaction d, here we assume to control the maximum value $m_{s,\max}^{(d)}$ for each order d, which still allows us to tune the differences in memory across different orders We consider a higher-order network of $N = 5$ nodes, composed by $T = 3 \cdot 10^5$ snapshots, constructed using the DARH model. We consider hyperedges up to order four. The maximum memory lengths for the interactions of orders two, three and four are set to $m_{s,\max}^{(2)} = 50$, $m_{s,\max}^{(3)} = 40$, and $m_{s,\max}^{(4)} = 15$, respectively. The probabilities of drawing from the past are set to $q^{(2)} = 0.60$, $q^{(3)} = 0.45$, and $q^{(4)} = 0.40$. Fig. S9 shows the resulting

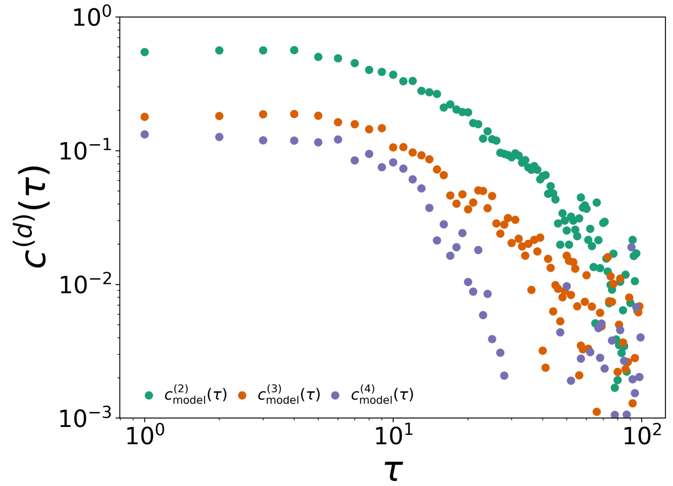

FIG. S9. Intra-order correlation functions in the DARH model with uniform distribution of memory. Each color represents a different order of interaction.

intra-order correlation functions for each order of interaction. Comparing the figure with Fig. S8(a), we can clearly appreciate the impact of heterogeneity in the hyperdge memory. When all hyperedge of order d are characterized by the same memory length $m_s^{(d)}$, we find that the intra-order correlation function remains constant for $1 \le \tau \le m_s^{(d)}$, while it decays exponentially after that value. Instead, when the hyperedge have different memories, the profiles of the temporal correlation functions $c^{(d)}$ resemble those observed in the data, with a slow decay followed by a loss of correlation.

IMPACT OF THE HYPERGRAPH SIZE IN THE DARH MODEL

In this section, we analyze the impact of the number of nodes on the temporal correlations existing in a temporal hypergraph generated with the DARH model. We build higher-order networks of $N = 10, 20, 30$ and 40 nodes, each one composed by $T = 10^5$ snapshots, considering interactions up to order four. In all cases, we set the memory lengths for the interactions of orders two, three and four to $m_s^{(2)} = 5$, $m_s^{(3)} = 4$, and $m_s^{(4)} = 3$, respectively, with the probabilities of drawing from the past set to $q^{(2)} = 0.60$, $q^{(3)} = 0.55$, and $q^{(4)} = 0.50$. The probabilities to activate each hyperedge in the memory-less process are set as a function of the number of nodes, particularly $y^{(2)} = 5/N$, $y^{(3)} = 1/N^2$, and $y^{(4)} = 1/N^3$, this way accounting for the fact that interactions of higher orders are less abundant in empirical systems [5].

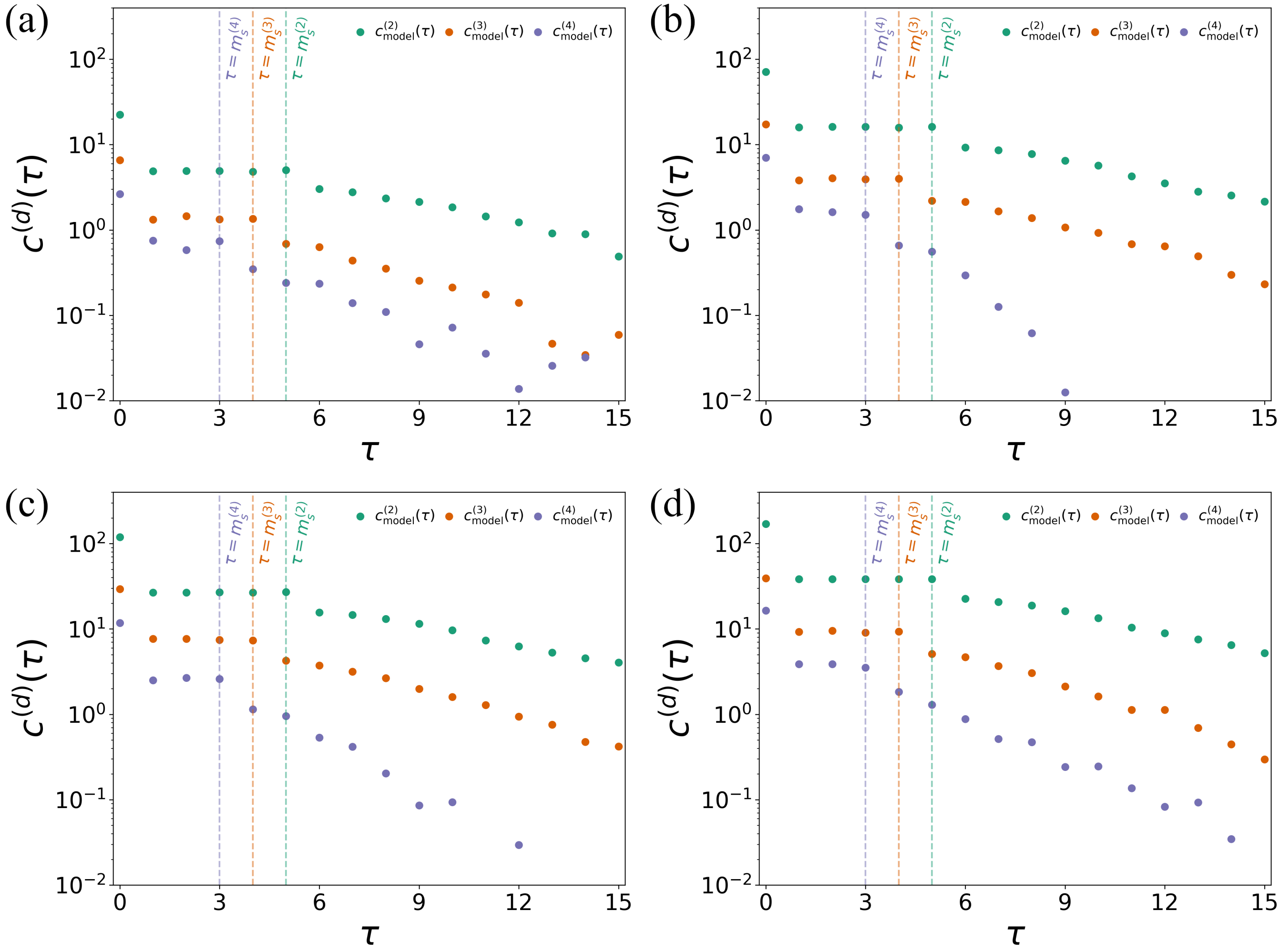

FIG. S10. Intra-order correlation functions in the DARH model as a function of the number of nodes. Panels (a) to (d) represent the cases of temporal hypergraphs with $N = 10, 20, 30$ and 40 nodes, respectively. Each color represents a different order of interaction. Vertical dashed lines represent the values $\tau = m_s^{(d)}$, with $d \in 1, 2, 3$.

Fig. S10 show the intra-order correlation functions evaluated for the four temporal hypergraphs. We observe that the qualitative behavior of the correlation functions does not change with the number of nodes. For each hypergraph size, the intra-order correlation remain constant until $\tau \leq m_s^{(d)}$ (vertical dashed lines), decaying exponentially after that value. The number of nodes, however, has an effect on the scale of $c^{(d)}$. Particularly, the larger the temporal hypergraph, the higher the value of the intra-order correlation function. This does not come as a surprise, as the correlation functions $c^{(d)}$ are defined as the trace of the correlation matrices $\mathcal{C}^{(d)}$ (see Eq. (2) in the main text), which is a square matrix of order N. These results probe the robustness of our analysis with regards to the number of nodes, showing that the emergence of temporal correlations in higher-order networks is not due to size effects.

ANALYSIS OF THE CDARH MODEL

In this section we further analyze the cross-memory Discrete Auto-Regressive Hypergraph (cDARH) model. As described in the main text, in the cDARH model, a hyperedge of order d can update its state by drawing from its previous states or from the previous states of a hyperedge of a different order d'. Using the cDARH model, we generate a temporal hypergraph with $N = 10$ nodes, maximum hyperedge order $D = 3$ and a temporal range of $T = 3 \cdot 10^4$ time steps. We set $p^{(2)} = 0$ and $p^{(3)} = 0.6$, meaning that hyperedges of order three can copy from the past of hyperedges of order two, while hyperedges of order two evolve independently. For the cross-order memory of 3-hyperedges, we set $m_c^{(2,3)} = 60$, while we set $m_s^{(2)} = 40$ and $m_s^{(3)} = 10$ for the intra-order memories of hyperedges of order two and three, respectively. Fig. S11(a) reveals the presence in hyperedges of order two (green circles) and

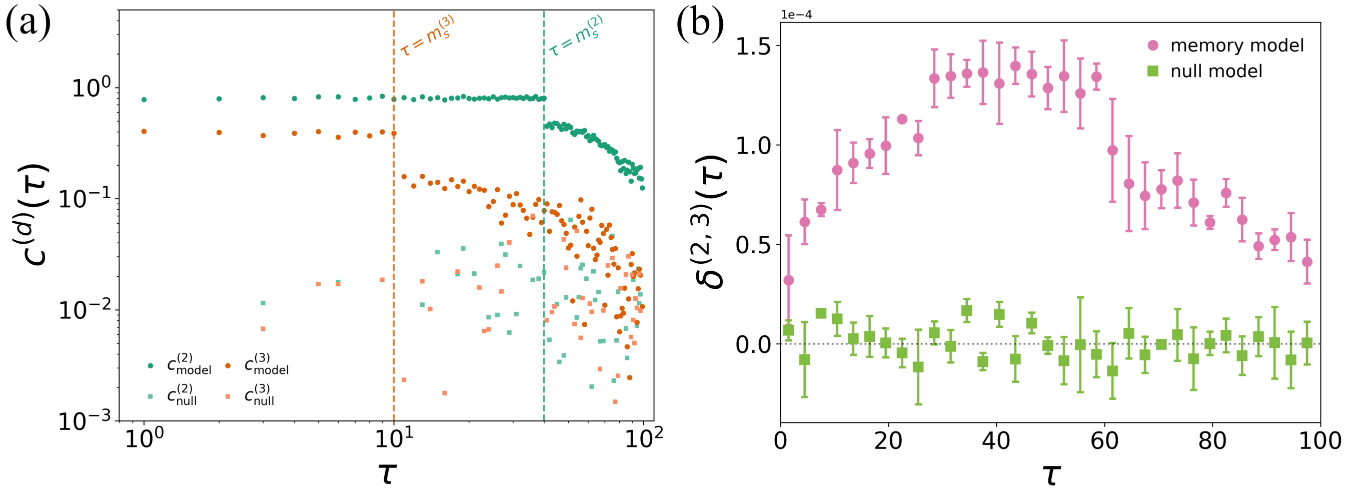

FIG. S11. Temporal correlations in the cDARH model. (a) Intra-order correlations $c^{(d)}$, with $d \in \{2, 3\}$, for hyperedges of order two (green circles) and three (orange circles). The dashed vertical lines correspond to the value of the intra-order memory of hyperedges of order two (green) and three (orange), respectively. (b) Cross-order gap function $\delta^{(2,3)}$ between hyperedges of order two and three (purple circles). We compare intra-order and cross-order correlations for the hypergraph generated using the cDARH model with a randomized null model (colored squares).

The values of the cross-order gap functions are binned averaged, with the error bars representing the standard deviation.

three (orange circles) of significant intra-order temporal correlations. In particular, we notice that the functions $c^{(2)}$ and $c^{(3)}$ remain constant for $\tau \leq m_s^{(d)}$, while rapidly decaying after that value. Though the profiles of $c^{(d)}(\tau)$ do not match exactly those of empirical data (see the main text), such a minimal model reveals that memory can be the driving mechanism for the emergence of intra-order temporal correlations, with different orders possessing different degrees of memory, explaining the hierarchical structure of correlation observed in the data.

Fig. S11(b) shows that $\delta^{(2,3)}(\tau) > 0$ for different values of τ (purple circles), meaning that hyperedges of order two are correlated to hyperedges of order three occurring later in time more than the other way around. Such a result suggests that cross-order memory is a fundamental factor for the emergence of cross-order correlations among different orders of interaction, as well as cross-order gaps in real-world social systems.

We now focus on the trend of the intra-order correlation functions. In this setting, we find that the intra-order correlation function $c^{(2)}(\tau)$ can be reproduced by the Yule-Walker equations (S2), while $c^{(3)}(\tau)$ can not. This is not surprising, as hyperedges of order two evolve independently, so their state follows a independent DAR process (see previous section for more detail), while the evolution of hyperedges of order three also depends on the dynamics of three overlapping hyperedges of order two.

To gain insights about the behavior of the cDARH model, we fit the exponential decay of both $c^{(2)}(\tau)$ and $c^{(3)}(\tau)$. We find that both exponential decays have the same rate, i.e., $c^{(d)} \sim e^{\beta\tau}$, with $\beta = 0.02$, as reported in Fig. S12. This result is essentially due to the fact that hyperedges of order three have memory of the overlapping hyperedges of order two. This mechanism might also explain why interactions in real-world systems are characterized by long-range intra-order correlation functions that decays with similar rates (see Fig. S1 and Fig. 1 in the main text). Note that, to better appreciate the rate comparison, here we consider a lin-log scale, whereas in Fig. 3(c) of the main text we used a log-log scale.

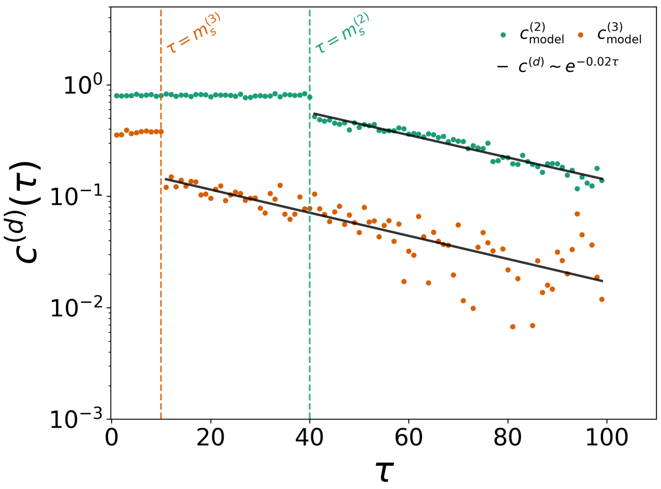

FIG. S12. Intra-order correlations in the cDARH model. Green and orange circles represent the value of $c^{(d)}$ for hyperedges of orders two and three, respectively. The dashed vertical lines correspond to the value of the intra-order memory of hyperedges of order two (green) and three (orange). The solid black line displays the best fit of the exponential decay for both $c^{(2)}$ and $c^{(3)}$.

CORRELATION ANALYSIS OF A PERIODIC TEMPORAL HYPERGRAPH

Social systems show a variety of temporal patterns, from periodicity to decaying autocorrelations. In the main text, we concentrated on this latter feature, discussing a minimal model of temporal higher-order networks with memory. In this section, we focus instead on temporal periodic patterns in systems characterized by higher-order interactions. To do so, we build a temporal hypergraph of N nodes where each order of interaction has different periodicity. For simplicity and with no loss of generality, we limit our analysis to groups of size two and three. First, we construct a sequence of length T_2 of random 2-uniform hypergraphs, namely hypergraphs where all hyperedges have order two. Each hyperedge has a probability p_2 to be created. Each hypergraph of the sequence is nothing more than a Erdös-Rényi random network, i.e., $\mathrm{ER}(N, p_2)$. We then construct another sequence, having length T_3, and composed by random 3-uniform hypergraphs, i.e., all hyperedges connect exactly three nodes. A hyperedge has a probability p_3 to be created. For each order of interaction, we concatenate several of the corresponding sequences one after another, thus building two temporal uniform hypergraphs. We repeat the sequences so that both temporal hypergraphs have T time-steps each. Finally, for each time-step we join the two corresponding static uniform hypergraphs. By doing this, we now have a single temporal hypergraph of length T where the 2-hyperedges are repeated every T_2 time-steps, while the 3-hyperedges have instead a period T_3.

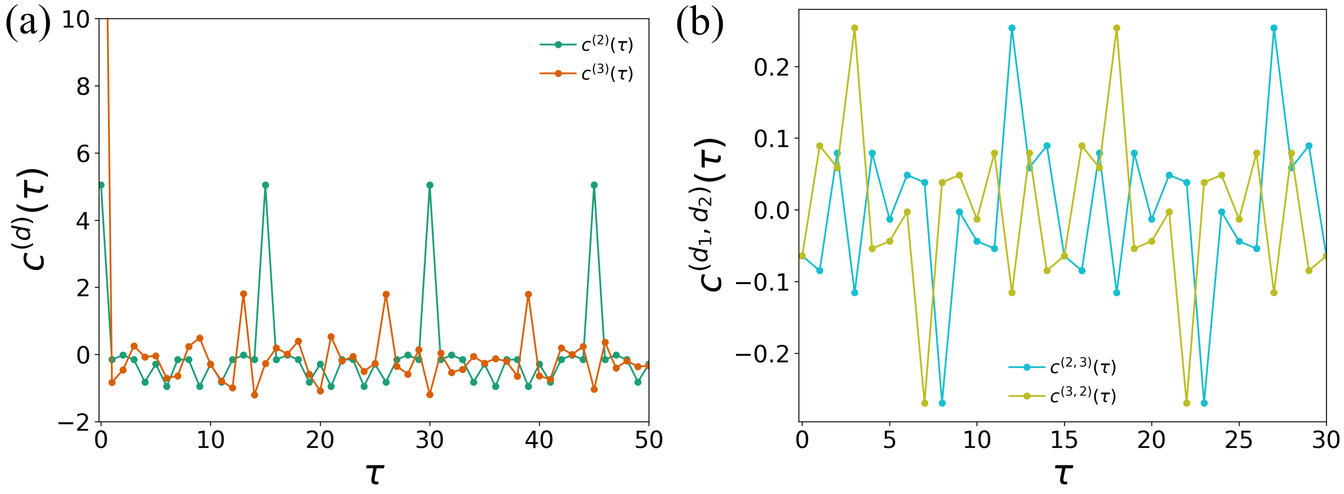

FIG. S13. Intra-order correlation functions and cross-order gap functions in a minimal model of periodic temporal hypergraphs. (a) Intra-order correlations in hyperedges of order two (green) and three (orange), respectively. (b) Cross-order correlation functions between hyperedges of orders two and three, i.e., $c^{(2,3)}(\tau)$ (cyan) and $c^{(3,2)}(\tau)$ (olive).

Fig. S13 shows the intra-order correlation functions (a) and the cross-order correlation functions (b) for a periodic temporal hypergraph of $N = 10$ nodes. The hypergraph is composed by $T = 1.95 \cdot 10^5$ snapshots. Each 2-hyperedge has a probability $p_2 = 0.1$ of existing and it repeats with period $T_2 = 15$. The 3-hyperedges have instead a probability $p_3 = 0.03$ of being active and they have period $T_3 = 8$.

Both orders of interactions clearly show a periodic behavior. Indeed, we observe that the intra-order correlation functions peak at $\tau = kT_2$ and $\tau = kT_3$, with $k \in \mathbb{N}$, for two-body and three-body interactions, respectively, while $c^{(2)}(\tau)$ and $c^{(3)}(\tau)$ remain close to zero for other values of τ. The periodicity of the temporal hypergraph is also reflected in the cross-order correlation functions $c^{(2,3)}(\tau)$ (cyan) and $c^{(3,2)}(\tau)$ (olive). In particular, we observe that they peak at different harmonics and that in general $c^{(2,3)}(\tau) \neq c^{(3,2)}(\tau)$, as the periodic oscillations of the two orders of interaction are not in phase.

SUPPLEMENTARY REFERENCES

[1] M. Génois, C. L. Vestergaard, J. Fournet, A. Panisson, I. Bonmarin, and A. Barrat, Data on face-to-face contacts in an office building suggest a low-cost vaccination strategy based on community linkers, Network Science **3**, 326 (2015).

[2] P. Vanhems, A. Barrat, C. Cattuto, J.-F. Pinton, N. Khanafer, C. Régis, B.-a. Kim, B. Comte, and N. Voirin, Estimating potential infection transmission routes in hospital wards using wearable proximity sensors, PloS one **8**, e73970 (2013).

[3] P. Sapiezynski, A. Stopczynski, D. D. Lassen, and S. Lehmann, Interaction data from the copenhagen networks study, Scientific Data **6**, 315 (2019).

[4] O. E. Williams, F. Lillo, and V. Latora, Effects of memory on spreading processes in non-markovian temporal networks, New Journal of Physics **21**, 043028 (2019).

[5] G. Cencetti, F. Battiston, B. Lepri, and M. Karsai, Temporal properties of higher-order interactions in social networks, Scientific reports **11**, 7028 (2021).
